# Discriminating radiation injury from recurrent tumor with [^18^F]PARPi and amino acid PET in mouse models

**DOI:** 10.1186/s13550-018-0399-z

**Published:** 2018-07-04

**Authors:** Patrick L. Donabedian, Susanne Kossatz, John A. Engelbach, Stephen A. Jannetti, Brandon Carney, Robert J. Young, Wolfgang A. Weber, Joel R. Garbow, Thomas Reiner

**Affiliations:** 1 Department of Radiology, Memorial Sloan-Kettering Cancer Center, 1275 York Avenue, New York, NY 10065 USA; 2 Department of Radiology, Washington University, St. Louis, MO USA; 3 Alvin J. Siteman Cancer Center, Washington University, St. Louis, MO USA; 4 Department of Chemistry, Hunter College of the City University of New York, New York, NY USA; 5 Ph.D. Program in Biochemistry, Graduate Center of the City University of New York, New York, NY USA; 6 Ph.D. Program in Chemistry, Graduate Center of the City University of New York, New York, NY USA; 7 Department of Radiology, Weill Cornell Medical College, New York, NY USA; 8 Molecular Pharmacology and Chemistry Program, Memorial Sloan-Kettering Cancer Center, New York, NY USA; 9 Department of Nuclear Medicine, Technical University Munich, Munich, Germany

**Keywords:** PET/CT, PARP1, Radiation necrosis, Radiation injury, Amino acid PET, Biomarkers

## Abstract

**Background:**

Radiation injury can be indistinguishable from recurrent tumor on standard imaging. Current protocols for this differential diagnosis require one or more follow-up imaging studies, long dynamic acquisitions, or complex image post-processing; despite much research, the inability to confidently distinguish between these two entities continues to pose a significant dilemma for the treating clinician. Using mouse models of both glioblastoma and radiation necrosis, we tested the potential of poly(ADP-ribose) polymerase (PARP)-targeted PET imaging with [^18^F]PARPi to better discriminate radiation injury from tumor.

**Results:**

In mice with experimental radiation necrosis, lesion uptake on [^18^F]PARPi-PET was similar to contralateral uptake (1.02 ± 0.26 lesion/contralateral %IA/cc_max_ ratio), while [^18^F]FET-PET clearly delineated the contrast-enhancing region on MR (2.12 ± 0.16 lesion/contralateral %IA/cc_max_ ratio). In mice with focal intracranial U251 xenografts, tumor visualization on PARPi-PET was superior to FET-PET, and lesion-to-contralateral activity ratios (max/max, *p* = 0.034) were higher on PARPi-PET than on FET-PET.

**Conclusions:**

A murine model of radiation necrosis does not demonstrate [^18^F]PARPi avidity, and [^18^F]PARPi-PET is better than [^18^F]FET-PET in distinguishing radiation injury from brain tumor. [^18^F]PARPi-PET can be used for discrimination between recurrent tumor and radiation injury within a single, static imaging session, which may be of value to resolve a common dilemma in neuro-oncology.

**Electronic supplementary material:**

The online version of this article (10.1186/s13550-018-0399-z) contains supplementary material, which is available to authorized users.

## Background

Treatment of glioblastoma generally proceeds with surgical debulking or resection followed by adjuvant chemotherapy, usually including temozolomide, and whole-brain or targeted radiation therapy [1]. A frequent and problematic development in the management of patients with brain cancer post-radiotherapy is the appearance of an increased or new enhancing lesion within the radiation field, which could be either a recurrent tumor or radiation injury [2]. Radiation injury spans a spectrum from the clinically defined “pseudoprogression,” which is early, self-limiting, and spontaneously resolving, to radiation necrosis, which is late and unrelenting. Radiation injury is a major, dose-limiting complication of therapeutic brain irradiation and the cause of significant cognitive symptoms and loss of quality-of-life. Radiation injury shares most salient image features with recurrence on computed tomography (CT) and most magnetic resonance imaging (MRI) sequences [3]. Development of radiation injury is often delayed weeks to months post-radiation therapy, mimicking the time course of a potential recurrent tumor, and may be due to radiation damage to vascular endothelial cells leading to ischemic injury and demyelinization [4], direct radiation-induced killing of oligodendrocytes [5, 6], or cytokine-mediated host immune response [7]. Crucially, protocols for clinical management of radiation necrosis and recurrent tumor are incompatible, making their differential diagnosis an important, yet unmet clinical need.

Positron emission tomography using [^18^F]fluorodeoxyglucose (FDG), [^18^F]fluorothymidine [8, 9], [^11^C]methionine [10, 11], [^11^C]choline [12], and MRI sequences such as diffusion-weighted imaging [13], perfusion imaging [14], MR spectroscopy [15], and fluid-attenuated inversion recovery [16] has been investigated to differentiate radiation injury from recurrent tumor in human patients with variable sensitivity and specificity. Current diagnostic protocols using MRI and FDG-PET require one or more follow-up imaging studies [2], delaying diagnosis and definitive choice of treatment.

**Fig. 1:**
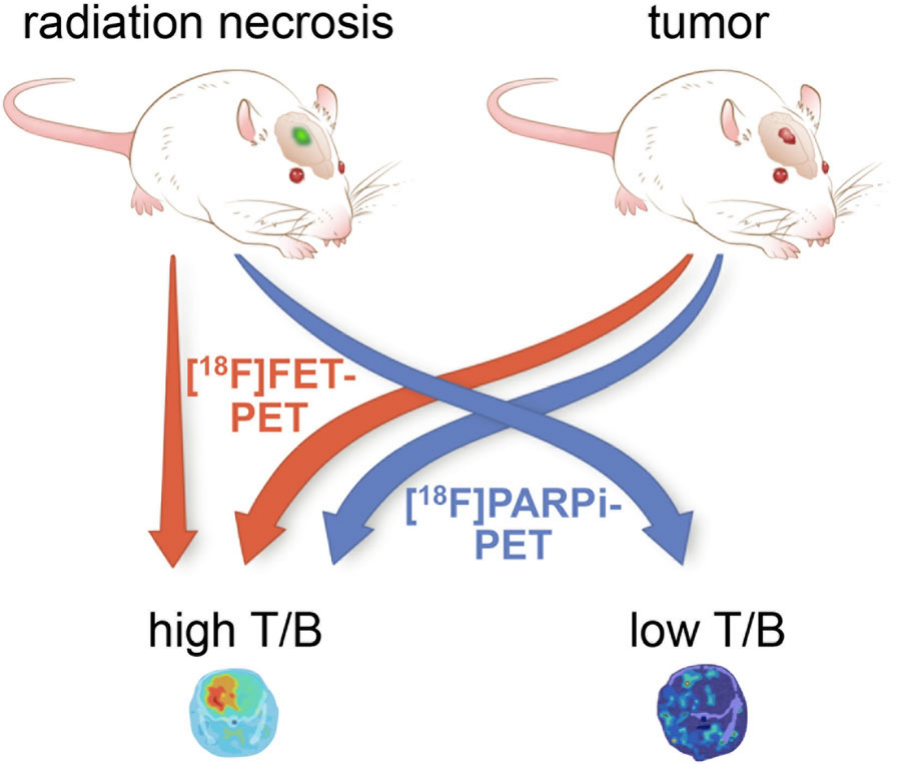
Experimental setup and hypothesis for imaging radiation necrosis and orthotopic brain tumors with [^18^F]FET-PET and [^18^F]PARPi-PET. Due to the molecular properties of each lesion and tracer, [^18^F]FET-PET will generate high lesion-to-background contrast in both tumor and radiation necrosis, while [^18^F]PARPi-PET will generate high contrast only in tumor

Poly(ADP-ribose) polymerase (PARP) is a family of nuclear enzymes associated with DNA-damage response that are highly expressed in many human cancers, including several with significant incidence, including breast [17], lung [18], ovarian, and others [19]. PARP overexpression may be a side effect of transformation, it may be an adaptation to high genomic instability [20], or it may drive tumorigenesis by inflammatory [21] or other mechanisms [22–24]. Therapeutic inhibition of PARP has met with success in the clinic, leading to FDA approval of three small-molecule PARP inhibitors (olaparib, rucaparib, and niraparib) between 2014 and 2017. As a molecular marker of cancer upregulated during tumor evolution, PARP-targeted imaging is a promising candidate for efficiently distinguishing neoplastic pathology from other conditions that share hallmarks of brain tumors, including enhancement and hypermetabolism [25]. Since radiation injury lacks the high proliferation and genomic instability that would drive, or be driven by, PARP overexpression, we theorized that radiation injury would not present with elevated PARP expression. Based on this physiological rationale, we formulated the hypothesis that PARPi-PET could accurately distinguish radiation injury from recurrent tumor, and would outperform amino acid PET in differentiating these two important clinical entities (Fig. 1). Two current-generation fluorine-18-labeled PET tracers exist for imaging PARP expression: [^18^F]PARPi [26] and [^18^F]FluorThanaTrace [27], with structural similarities to olaparib and rucaparib, respectively.

Amino acid PET was selected as an imaging agent for comparison to [^18^F]PARPi-PET because of multiple prior human and animal studies in differentiating radiation injury from tumor and the strong molecular rationale for its effectiveness in this setting [28–30]. To support the metabolic demands of proliferation and invasion, cancers may overexpress various solute transporters to draw useful substrates into the cell. Among these is the CD98-LAT1 heterodimeric amino acid transporter, which facilitates transmembrane diffusion of large, neutral amino acids in a sodium-independent manner [31, 32]. Among radiotracers reasonably specific for the LAT1 transport system [33, 34], [^18^F]FET was chosen due to its relatively straightforward synthesis.

Here, we present preclinical data showing that experimental murine radiation necrosis does not present with elevated levels of PARP1 expression and is not [^18^F]PARPi-avid in vivo. We further show that [^18^F]PARPi-PET outperforms [^18^F]FET-PET in distinguishing radiation injury from focal intracranial xenografts.

## Methods

### General

All animal experiments were performed in accordance with protocols approved by the Institutional Animal Care and Use Committees of Memorial Sloan Kettering Cancer Center (MSK) or Washington University and followed the National Institutes of Health guidelines for animal welfare. 4-(4-Fluoro-3-(piperazine-1-carbonyl)benzyl)phthalazin-1(2*H*)-one [35] and 4-(4-fluoro-3-(4-(4-fluorobenzoyl)piperazine-1-carbonyl)benzyl)phthalazin-1(2*H*)-one [26] ([^19^F]PARPi) were synthesized following the original literature procedures. Other chemicals were procured from commercial suppliers and used without further purification. 4,7,13,16,21,24-Hexaoxa-diazabicyclo[8.8.8]hexacosane (K_222_), dry dimethyl sulfoxide (DMSO) over molecular sieves, dry acetonitrile over molecular sieves, ethyl 4-nitrobenzoate, and miscellaneous chemicals were purchased from Sigma-Aldrich (St. Louis, MO). l-tyrosine, *O*-(2-tosyloxyethyl)-*N*-trityl, tert-butyl ester, and (*O*-(2-fluoroethyl)-l-tyrosine) hydrochloride ([^19^F]FET) were purchased from ABX (Radeberg, Germany). High-performance liquid chromatography (HPLC) purification and analysis was performed on a Shimadzu UFLC HPLC system with a DGU-20A degasser, an SPD-M20A UV detector, an LC-20AB pump unit, and a CBM-20A communication bus module, with a LabLogic Scan-RAM radio-TLC/radio-HPLC detector setup to detect radioactive signal. All averages are presented as mean ± standard deviation.

### Radiochemistry

No-carrier-added [^18^F]fluoride was produced by the ^18^O(p,n)^18^F reaction of 16.5 MeV protons incident on an ^18^O-enriched water target in a GE Healthcare PETTrace 800 cyclotron. To separate the [^18^F]fluoride in a form suitable for nucleophilic fluorination, the ^18^O-enriched water containing [^18^F]fluoride was passed through an anion-exchange cartridge (Waters Sep-Pak QMA Light), which was then eluted with 2 mL of 11.95 mM K_222_ and 20 mM K_2_CO_3_ in 4% H_2_O/acetonitrile into a kiln-dried V-vial. Water was removed azeotropically by heating at 120 °C under a gentle stream of nitrogen until almost no visible liquid remained; further rounds of azeotropic drying were unnecessary and did not affect radiochemical yields or molar activity.

Radiosynthesis of [^18^F]PARPi was completed as described previously, using an optimized three-step, one-pot reaction with a prosthetic group (Additional file 1: Figure S1a). Briefly, [^18^F]4-fluorobenzoic acid was prepared by fluorination of ethyl 4-nitrobenzoate (500 μg) with [^18^F]KF-K_222_ at 120 °C in dry DMSO (100 μL), followed by hydrolysis of the ethyl ester by addition of 50 μL of 1 M NaOH, followed by neutralization with 50 μL of 1 M HCl. HBTU (10 mg), triethylamine (20 μL), and 4-(4-fluoro-3-(piperazine-1-carbonyl)benzyl)phthalazin-1(2*H*)-one (2 mg) were added to the reaction vessel along with 100 μL DMSO and stirred at room temperature for 30 s. After adding 400 μL acetonitrile and 1 mL water, the crude reaction was purified on semi-preparative C6-phenyl reversed-phase HPLC using a 7:3 water/acetonitrile isocratic method (Waters Gemini C6-Phenyl, 5 μm, 10 × 250 mm, 5 mL/min). The collected HPLC peak (31–32 min, 5–6 mL) was diluted 10:1 with water, trapped on a C18 Sep-Pak Light cartridge, eluted with 400 μL of ethanol, and diluted in sterile normal saline, affording [^18^F]PARPi in consistent, acceptable radiochemical yield (22 ± 11%, not decay corrected), radiochemical purity (99%), and molar activity (> 15 GBq/μmol). Chemical identity of the radiopharmaceutical was confirmed by co-elution on HPLC with an analytically identified nonradioactive [^19^F]PARPi standard (Additional file 1: Figures S2 and S3).

Radiosynthesis of [^18^F]FET was completed as described in the literature [36], modifying the procedure slightly to accommodate manual synthesis (Additional file 1: Figure S1b). Briefly, a commercially available precursor, 6 mg of l-tyrosine, *O*-(2-tosyloxyethyl)-*N*-trityl, tert-butyl ester (ABX, Radeberg, Germany) was fluorinated directly by heating at 85 °C with [^18^F]KF-K_222_ in 500 μL of dry acetonitrile. Solvent was removed under gentle nitrogen flow at 85 °C, followed by removal of protecting groups on the amino acid moieties by heating at 75 °C with 1 mL of a 1:2 mixture of trifluoroacetic acid and 1,2-dichloroethane. After cooling to room temperature and adding 3 mL dichloromethane, the labeled amino acid was separated from organic components by trapping it on a silica cartridge (Waters Sep-Pak Silica Light). The cartridge was washed with 5 mL of 1:1 diethylether/hexanes, dried with nitrogen for 3 min, and then eluted with glycine buffer (2 mL, 0.5 M, pH 9.5) and the reaction product purified on semi-preparative C18 reversed-phase HPLC using a 2:98 ethanol/water isocratic method (Waters Gemini C18, 5 μm, 10 × 250 mm, 3 mL/min). The collected HPLC peak (22–24 min, 6–7 mL) was diluted in sterile normal saline, affording [^18^F]FET with good radiochemical yield (43 ± 22%, not decay corrected), radiochemical purity (99%), and molar activity (> 15 GBq/μmol). Chemical identity of the radiopharmaceutical was confirmed by co-elution on HPLC with an analytically identified nonradioactive standard [^19^F]FET (Additional file 1: Figures S2 and S3).

### Cell culture

U251 cells were kindly provided by the laboratory of Dr. Ronald Blasberg at MSK. U251 cells were maintained in 150 cm^2^ tissue culture flasks in Eagle’s modified essential medium supplemented with 10% heat-inactivated fetal bovine serum, 100 IU/mL penicillin, and 100 μg/mL streptomycin, stored in a cell culture incubator at 37 °C and 5% CO_2_ atmosphere, changing media every 2 days and passaging at 70% confluence. Validation of cell line identity by STR fingerprinting was performed by MSK’s Integrated Genomics Core (Additional file 1: Table S1).

### Animal models

Mice were irradiated at Washington University. To generate mice with experimental radiation necrosis, 6- to 8-week-old, female BALB/c mice (*n* = 10; Harlan; Indianapolis, IN) were irradiated with the Leksell Gamma Knife (GK) Perfexion™ (Elekta AB; Stockholm, Sweden), as described previously [37]. Mice were anesthetized with a mixture of ketamine (25 mg/kg) and xylazine (5 mg/kg), injected intra-peritoneally 5 min before the start of irradiation, supported on a specially designed platform mounted to the stereotactic frame that attaches to the treatment couch of the instrument, and 50 Gy (at 50% isodose) in a single fraction was delivered to the left cerebral hemisphere. This treatment induces radiation necrosis on an experimentally tractable time scale (progressing over 4–12 weeks following irradiation) in BALB/c mice [37].

To generate mice with intracranial U251 tumors, 6-to 8-week old, outbred female homozygous athymic nude mice (Foxn1^nu/nu^; *n* = 10; ENVIGO, East Millstone, NJ) were anesthetized with 2% isoflurane in 2 L/min medical air and placed in the frame of a Stoelting Digital New Standard stereotactic device (Stoelting, Wood Dale, IL) on a rodent warming pad with maintenance anesthesia delivered by nose cone. A paramedian scalp incision was created, and subperiosteal dissection exposed the anterior part of the coronal and sagittal sutures. Using only the beveled tip of a sterile 25-G needle, a craniotomy was created 2 mm lateral and 1 mm anterior to the bregma. A 5-μL Hamilton syringe with a 28-G flat tip needle, mounted to the stereotactic arm, was gently inserted through the craniotomy to a depth of 3 mm, and U251 cells (5 × 10^5^ in 1.5 μL of sterile phosphate-buffered saline) were injected slowly over 5 min. After completion of the injection, the syringe was left in place for an additional 5 min to allow intracranial pressure to equalize, preventing reflux of the cells out of the craniotomy and the formation of extracranial tumor.

### Imaging

Brain MRI scans were performed on a 7-T small animal MRI scanner (Bruker Biospin Corp., Billerica, MA) equipped with a 12-cm inner diameter gradient (Resonance Research Inc., Billerica, MA) with 640 mT/m maximum gradient amplitude. A custom-built 32-mm quadrature radiofrequency (RF) body coil (Stark Contrast MRI Coils Research, Erlangen, Germany) was used for RF excitation and detection with Bruker Avance electronics. The animals were anesthetized by 2% isoflurane in oxygen, and animal respiration during MRI was monitored by a physiological monitoring system (SA Instruments, Stony Brook, NY). For positioning, gradient-echo scout images of the brain along three orthogonal orientations were first acquired. The brain T2-weighted rapid acquisition with refocused echoes (RARE) axial scan was performed with 22 slices with 0.7-mm slice thickness covering the whole brain, repetition time (TR) = 2.37 s, echo time (TE) = 50 ms, 88 × 140 μm^2^ in-plane resolution, and approximately 14 min of acquisition. For the Gd-enhanced scan, Magnevist (Bayer Healthcare, Wayne, NJ) at a dose of 0.1 mmol/kg was injected via tail vein. Fast low-angle shot (FLASH) gradient-echo images were acquired immediately every 3 min for 15 min after the injection with the following parameters, TR = 130 ms, TE = 2.4 ms, 0.7-mm slice thickness, and 117 × 117 μm^2^ in-plane resolution. To determine contrast-enhancing area in tumor mice, the transaxial slice DCE-MR image with the largest contrast-enhancing area was loaded into the Fiji distribution of ImageJ [38] and a polygonal ROI drawn over the contrast-enhancing region.

For PET/CT imaging, mice were injected with radiotracer (7.4–18.5 MBq) through the lateral tail vein 2 h before imaging. PET/CT imaging was performed under inhaled isoflurane anesthesia (2% in medical air by precision vaporizer) on a Siemens Inveon PET/CT scanner (Siemens, Germany). After acquisition (5–15 min with > 20 million counts/scan), list mode emission data were sorted into two-dimensional sinograms via Fourier rebinning. Data were normalized to correct for non-uniform detector response, dead time count losses, and positron branching ratio, but no attenuation, scatter, or partial-volume averaging corrections were applied. Sinogram data were subsequently reconstructed into 128 × 128 × 159 matrix (0.78 × 0.78 × 0.80 mm^3^ voxel dimensions) using 2D ordered subset expectation maximization (OSEM2D; 4 iterations, 16 subsets). Image counts per voxel per second were converted to activity concentrations (Bq/cc or %IA/cc) using a system-specific calibration factor derived from imaging a mouse-sized water-equivalent phantom containing fluorine-18. CT scans were reconstructed using a modified Feldkamp cone beam reconstruction algorithm to generate 512 × 512 × 768 voxel image volumes (0.197 × 0.197 × 0.197 mm^3^ voxel dimensions). PET/CT images were processed using Inveon Research Workplace software, with a spherical VOI with a volume of 50–150 mm^3^ drawn in the lesion and in an unlesioned area of the contralateral hemisphere. To prevent partial volume effects suppressing apparent uptake in animals with very small tumors, tumor mice with contrast-enhancing regions below 1.5 mm^2^ on DCE-MR (*n* = 3 of 10) were excluded from PET data analysis.

### Autoradiography

Mice were sacrificed immediately after imaging by CO_2_ asphyxiation and cervical dislocation. Brains were dissected by removing the calvarium and gently dissecting the brain from the lower skull with the tip of a pair of forceps. Brains were embedded in OCT medium (Fisher Scientific, Houston, TX) on dry ice and sectioned at 10-μm thickness onto glass slides on a cryostatic microtome. Slides were placed into a cassette pressed against the pre-blanked storage phosphor plate, separated by a layer of plastic wrap, and the plate allowed to charge for ten half-lives at − 20 °C. The phosphor plate was read on a Typhoon FLA 7000 scanner (GE Healthcare, Port Washington, NY), and the slides were subjected to hematoxylin and eosin staining and scanned on a Mirax slide scanner (Carl Zeiss, Jena, Germany). Images were processed using Pannoramic Viewer (3DHISTECH Ltd., Budapest, Hungary) and the Fiji ImageJ distribution.

### Immunohistochemistry

Mice were sacrificed by CO_2_ asphyxiation, followed by intracardiac perfusion with phosphate-buffered saline and formalin. Brains were then dissected and fixed in 4% paraformaldehyde for 24 h before dehydration in 70% ethanol overnight, embedding in paraffin and sectioning. For PARP1 IHC, staining was performed using a Discovery XT processor (Ventana Medical Systems, Tucson, AZ). Formalin-fixed, paraffin-embedded 3-μm sections were deparaffinized with EZPrep buffer, antigen retrieval was performed with CC1 buffer (Ventana Medical Systems, Tucson, AZ), and sections were blocked for 30 min with Background Buster solution (Innovex, Richmond, CA). The tissue was incubated with anti-PARP1 rabbit polyclonal antibody (sc-7150, Santa Cruz Biotechnology, Santa Cruz, CA) for 5 h (0.2 μg/ml), followed by 1 h incubation with biotinylated goat anti-rabbit IgG (PK6106, Vector Labs, Burlingame, CA) at a 1:200 dilution. For detection, a DAB detection kit (Ventana Medical Systems, Tucson, AZ) was used according to the manufacturer’s instructions. Sections were counterstained with hematoxylin and coverslipped with Permount (Fisher Scientific, Pittsburgh, PA, USA). Slides were scanned on a Mirax slide scanner (Carl Zeiss, Jena, Germany).

## Results

### Mouse models

Irradiated mice developed lateral contrast-enhancing hyperintense regions in the dosed hemisphere on T2- and DCE-MR imaging, corresponding to radiation necrosis-induced edema, by 5 weeks post-irradiation (Additional file 1: Figure S4). At 9 weeks post-irradiation, edematous regions had expanded to cover > 60% of the irradiated hemisphere (Additional file 1: Figure S4) and mice developed stress and neurological symptoms (hunching, ataxia, poor grooming). By week 10, two mice (20%) died spontaneously; other mice were sacrificed over the course of the imaging experiments. Mice bearing intracranial U251 xenografts developed similar lateral contrast-enhancing hyperintense regions in the engrafted hemisphere on T2- and DCE-MR imaging, corresponding to edema from disordered tumor neovasculature. Tumor mice became moribund at 10–12 weeks post-engraftment.

### Immunohistochemistry

**Fig. 2:**
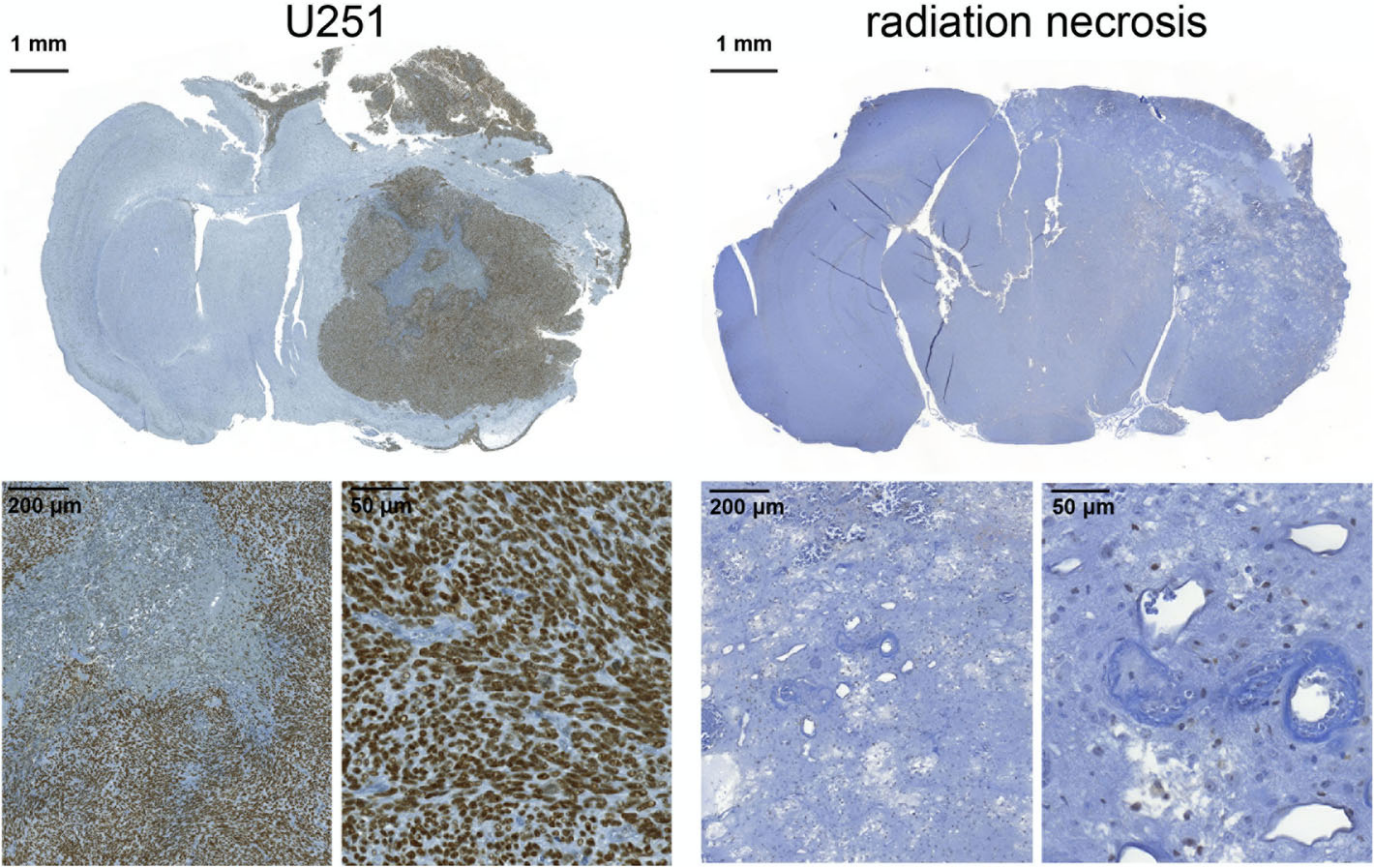
Anti-PARP1 immunohistochemistry. Staining of transaxial formalin-fixed, paraffin-embedded sections of mice implanted with U251 tumors (left group) mice with experimental radiation necrosis (right group) reveals high PARP1 expression in the nuclei of tumor cells and low PARP1 expression elsewhere in healthy brain and radiation necrosis

Immunohistochemistry indicates that radiation necrosis lesions and healthy brain express minimal PARP1, whereas U251 tumors overexpress PARP1 in the nuclei of tumor cells (Fig. 2).

### Autoradiography

**Fig. 3:**
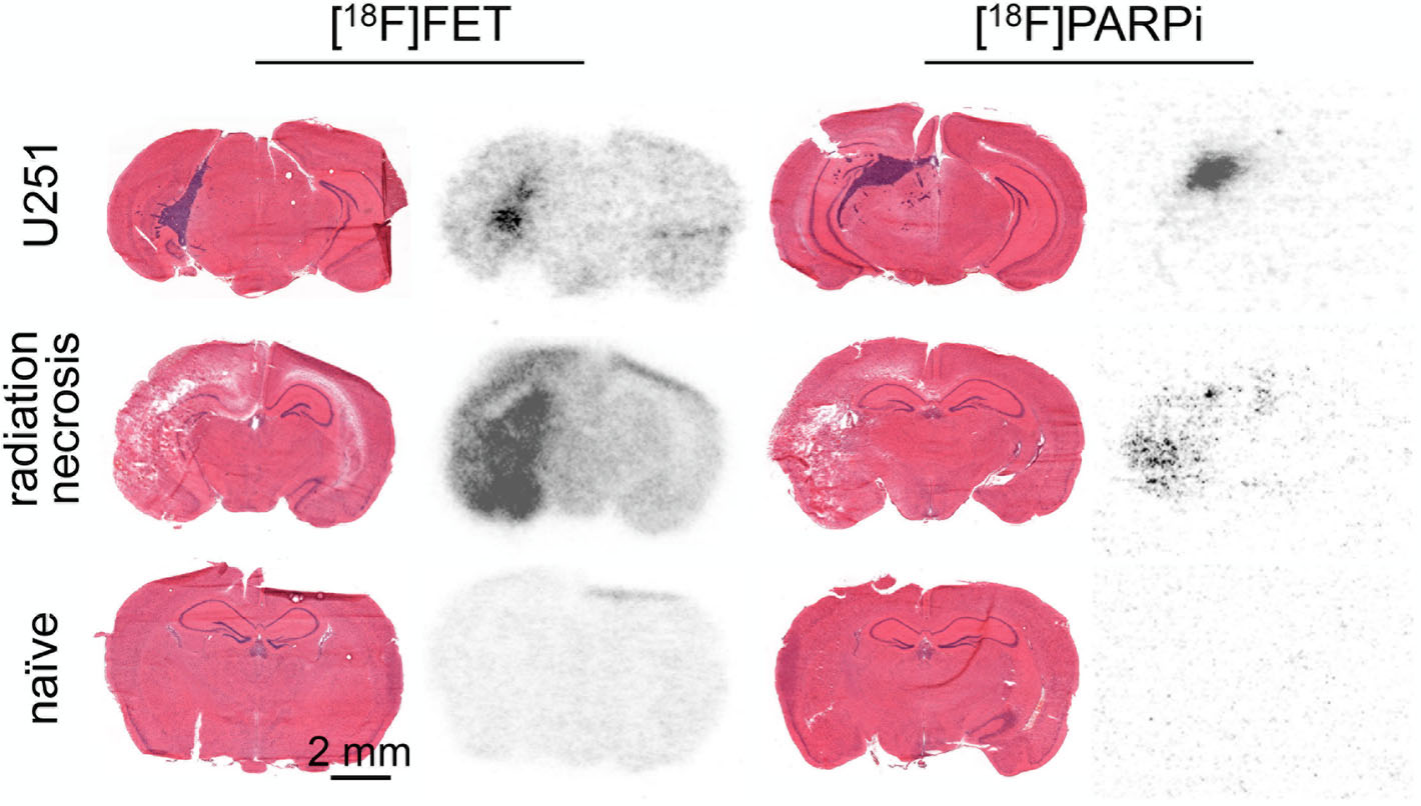
Hematoxylin and eosin stains and autoradiography. Hematoxylin and eosin stains (left) and digital storage phosphor autoradiography (right) of whole transaxial sections of mice with implanted U251 tumors (top row), experimental radiation necrosis (middle row), and naïve mice (bottom row), injected with [^18^F]FET (left column) or [^18^F]PARPi (right column). Autoradiographic scans have been contrast-adjusted for visibility. Mice were sacrificed 2.5 h post-injection of radiotracer

Storage phosphor autoradiography of cryosectioned tissue from animals injected with [^18^F]PARPi (11.1–18.5 MBq in 200 μL 5% ethanol in normal saline) and [^18^F]FET (11.1–18.5 MBq in 200 μL < 1% ethanol in normal saline) showed differential accumulation of both tracers in radiation necrosis and tumor (Fig. 3). In radiation necrosis animals, both [^18^F]PARPi and [^18^F]FET uptake were present on the lesioned side, but at much lower levels for [^18^F]PARPi. In U251 tumor animals, [^18^F]PARPi uptake was negligible in non-tumor areas but high in tumor areas, while [^18^F]FET uptake was moderate in non-tumor areas and high in tumor areas. Regions of tracer uptake co-localized with tumor (region of abnormal cellularity on H&E) or necrosis (region of tissue loss and disorganization on H&E). In treatment naïve animals, [^18^F]PARPi uptake was near-baseline and [^18^F]FET uptake was low but present.

### PET imaging

**Fig. 4:**
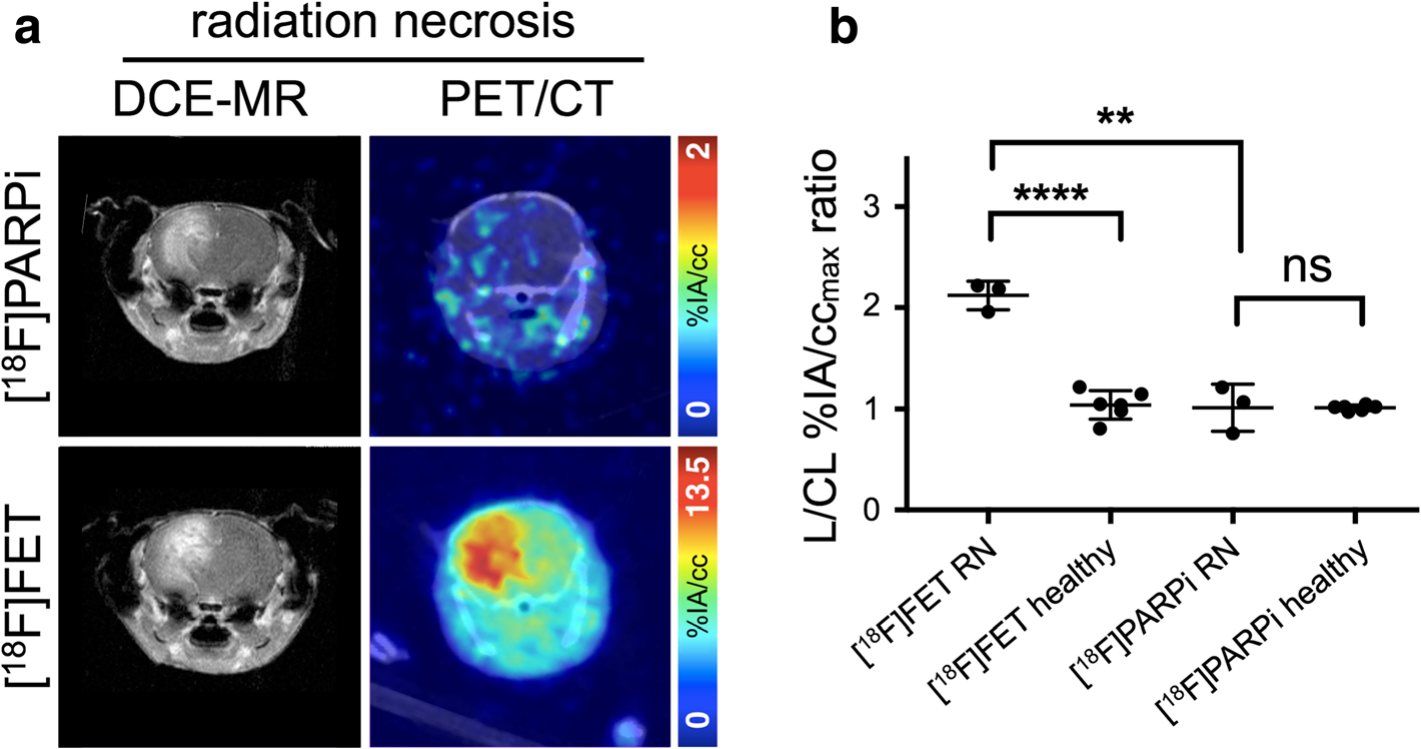
PET imaging of experimental murine radiation necrosis. **a** (left column) DCE-MR and (right column) fused PET/CT transaxial slices of mice with experimental radiation necrosis, injected with (top row) [^18^F]PARPi and (bottom row) [^18^F]FET. **b** Lesioned-to-contralateral hemisphere %IA/cc_max_ ratios for mice in different groups. **Significant at *p* < 0.005; ****significant at *p* < 0.0001

Complete tabular data for PET VOIs are available in Additional file 1: Tables S2 through S5. In mice with experimental radiation necrosis, lesion maximum voxel uptake on [^18^F]PARPi-PET was not higher than contralateral uptake (ratio: 1.02 ± 0.26 lesion/contralateral %IA/cc_max_ ratio; Fig. 4b), while [^18^F]FET-PET maximum voxel uptake corresponded to the contrast-enhancing region on MR with an uptake ratio of 2.12 ± 0.16 (lesion/contralateral %IA/cc_max_ ratio; Fig. 4b, Additional file 1: Figure S6). In mice with experimental radiation necrosis, lesion mean uptake on [^18^F]PARPi-PET was somewhat higher than contralateral uptake (ratio: 1.47 ± 0.11 lesion/contralateral %IA/cc_mean_ ratio; Additional file 1: Figure S7a), while [^18^F]FET-PET mean uptake ratios were much higher at 2.46 ± 0.39 (lesion/contralateral %IA/cc_mean_ ratio; Additional file 1: Figure S7a). [^18^F]FET-PET maximum voxel uptake ratios were significantly different between irradiated and treatment-naïve mice (*p* < 0.0001, unpaired *t* test; Fig. 4b), while [^18^F]PARPi-PET maximum voxel uptake ratios were not significantly different between irradiated and untreated mice (*p* = 0.98, unpaired *t* test; Fig. 4b; more images in Additional file 1: Figure S6).

**Fig. 5:**
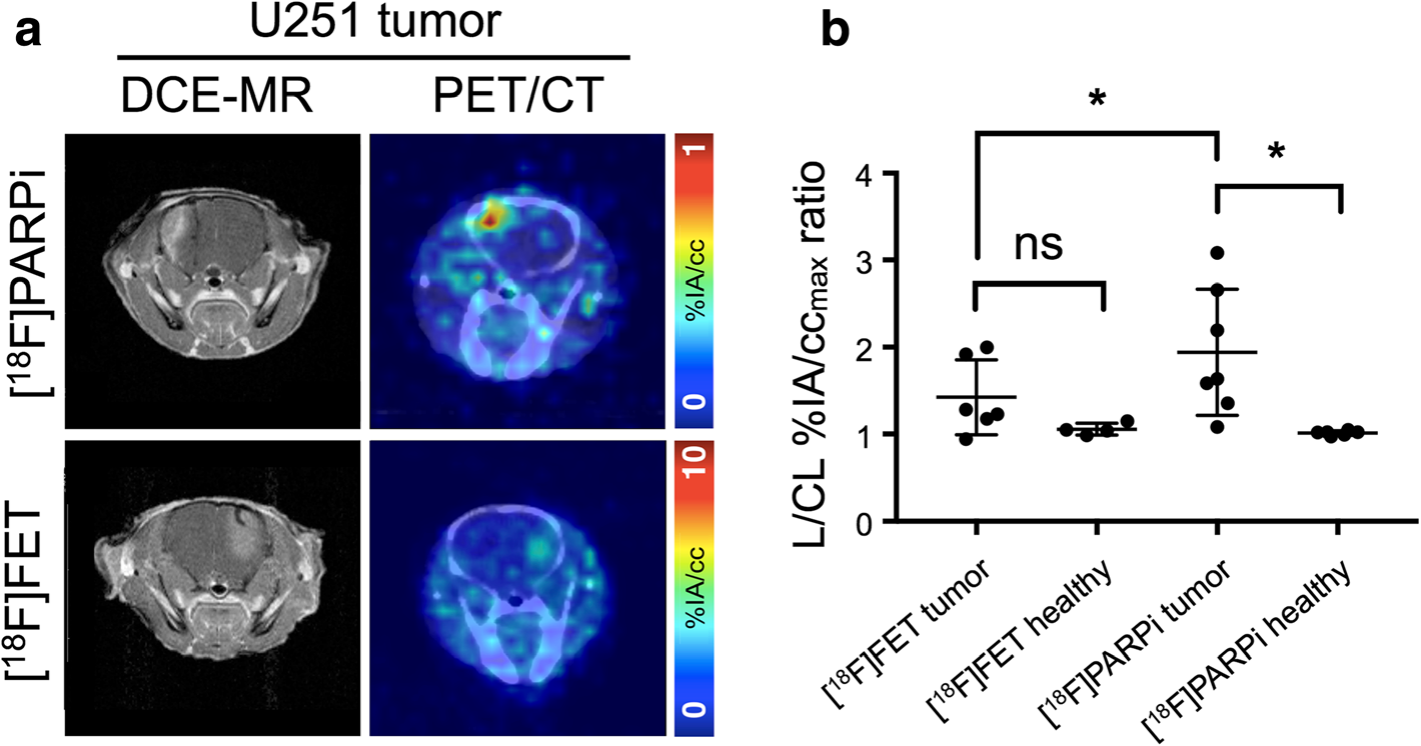
PET imaging of mice with focal intracranial U251 cell xenografts. **a** (left column) DCE-MR and (right column) fused PET/CT transaxial slices of mice with U251 tumor, injected with (top row) [^18^F]PARPi and (bottom row) [^18^F]FET. **b** Lesioned-to-contralateral hemisphere %IA/cc_max_ ratios for mice in different groups. *Significant at *p* < 0.05

In mice-bearing focal intracranial U251 xenografts, both [^18^F]PARPi and [^18^F]FET showed tumor accumulation, which varied widely according to tumor size (Fig. 5, Additional file 1: Figure S6). Contrast-enhancing areas on DCE-MR were correlated to tumor-to-background %IA/cc_max_ ratios on [^18^F]PARPi-PET and [^18^F]FET-PET (Pearson’s *r* > 0.95, Additional file 1: Figure S8 and Table S6). Visual delineation of the tumor from background was much easier on [^18^F]PARPi-PET than [^18^F]FET-PET. Quantification of the lesion/contralateral %IA/cc_max_ ratios showed higher ratios on [^18^F]PARPi-PET than [^18^F]FET-PET, for animals imaged on consecutive days with both tracers (*p* = 0.028, paired *t* test; Fig. 5b).

On PET imaging, [^18^F]PARPi uptake was observed in gut, liver, spleen, submandibular lymph nodes, and tumor, consistent with earlier reports [26]. [^18^F]FET uptake was observed in liver and bladder and systemically in muscle, radiation necrosis, and tumor lesions. Earlier reports have investigated [^18^F]FET uptake in radiation injury and brain tumors as well [30, 39, 40]. Notably, we found that systemic [^18^F]FET uptake was much higher in both tumor and radiation necrosis mice than in healthy BALB/cJ or nu/nu mice (Additional file 1: Figure S5).

## Discussion

Discrimination of radiation injury from recurrent or novel tumor in the brain within a single visit represents an urgent clinical need. Based on the theory that PARP overexpression is largely neoplasia-specific in adults, we hypothesized that radiation injury would not present with elevated levels of PARP1 expression. Consequently, radiation injury should not be significantly [^18^F]PARPi-avid (Fig. 1). Immunohistochemistry bore out the first hypothesis, showing that in an experimental model of radiation necrosis, mice did not develop elevated PARP1 expression as compared to contralateral or treatment-naïve brain, while glioblastoma xenografts expressed high levels of PARP1 in tumor cell nuclei (Fig. 2). This finding is consistent with studies in pediatric diffuse intrinsic pontine glioma [41] and adult glioblastoma [42], both of which found enhanced PARP1 expression in clinical samples of human brain tumors.

Our second hypothesis was confirmed by PET imaging: [^18^F]PARPi-PET did not reveal significant lateral uptake in radiation necrosis mice as compared to healthy controls despite BBB disruption, while [^18^F]FET-PET was strongly accumulated by radiation necrosis in our model. Furthermore, [^18^F]PARPi tumor to non-tumor ratios were significantly higher than [^18^F]FET tumor to non-tumor ratios in intracranial U251 xenografts.

The physiological basis for the differences in uptake between [^18^F]PARPi and [^18^F]FET is complex and suited to further investigation. Human clinical samples of radiation necrosis were shown in one study not to express LAT1 or its cofactor CD98 outside of endothelial cells and the occasional reactive astrocyte, while brain cancers highly express both factors at the tumor cell membrane [43]. Innate immunity [44] and immune cell infiltration [45, 46] are known processes in radiation injury of the brain and may be contributing to amino acid uptake from blood. Although various immune cells have shown to exhibit increased system l-mediated amino acid uptake under stimulated conditions [47–49], several studies demonstrated no increase in [^18^F]FET uptake in animal models of inflammation [50–52]. Perhaps more likely, low-affinity interactions may increase the residence time of tracers in cellular or extracellular compartments, causing [^18^F]FET to build up in the necrotic lesion. Therefore, both [^18^F]FET and [^18^F]PARPi uptake in our radiation necrosis model could be influenced by perfusion and blood volume, while [^18^F]FET uptake in radiation necrosis may additionally be transporter-mediated due to infiltration of the lesion by immune cells. Furthermore, [^18^F]PARPi’s nuclear target is present at high levels in the tumor lesion and almost completely absent in both healthy tissue and the necrotic lesion. The role of the PGP1 (also known as MDR1) multidrug efflux transporter is also potentially important in improving [^18^F]PARPi contrast in healthy brain regions. Expressed on the apical membrane of brain capillary endothelial cells, PGP1 regulates transcellular transport of a wide range of hydrophobic and amphipathic solutes [53]. [^18^F]PARPi is an olaparib analogue with a cyclopropamide moiety replaced with a parafluorobenzamide, and olaparib is a substrate of PGP1, with expression of PGP1 being a mechanism for olaparib resistance in tumor cells [54]. In both contrast-enhancing and non-contrast-enhancing regions, PGP1 activity may improve clearance of unspecifically bound material by removing drug that is not being retained by its target. Additionally, disruption of the BBB will reduce PGP1 activity and further enhance the specific retention of [^18^F]PARPi in PARP1 expressing brain lesions.

## Conclusions

Using mouse models of radiation necrosis and glioblastoma, we have demonstrated that PET imaging with [^18^F]PARPi can distinguish between these entities. The physicochemical properties of [^18^F]PARPi combine to reduce its uptake in regions, both necrotic and healthy, where its target is not expressed. Furthermore, PARP overexpression as a biomarker in the developed brain may be more specific to neoplasia than hypermetabolism of [^18^F]FET. Potentially efficient discrimination between recurrent tumor and radiation injury represents an added value for PARPi-PET and adds to the body of evidence supporting future clinical translation of this tracer.

## Additional file

Additional file 1: **Figures S1**–**S9** and **Tables S1**–**S6.** (PDF 14174 kb)

## Abbreviations

%IA/cc: Percent injected activity per cubic centimeter
%IA/g: Percent injected activity per gram
CT: Computed tomography (X-ray)
DCE-MR: Diffusion contrast-enhanced magnetic resonance imaging
DMSO: Dimethyl sulfoxide
DNA: Deoxyribonucleic acid
FDA: US Food and Drug Administration
FDG: Fluorodeoxyglucose
FLASH: Fast low-angle shot
HPLC: High-performance liquid chromatography
IHC: Immunohistochemistry
PARP: Poly(ADP-ribose) polymerase
PET: Positron emission tomography
RARE: Rapid acquisition with refocused echoes
RF: Radiofrequency
ROI: Region of interest
TE: Echo time
TR: Repetition time
VOI: Volume of interest
