# Supplementary material for: Discriminating radiation injury from recurrent tumor with [18F]PARPi and amino acid PET in mouse models
Source: EJNMMI Res. 2018 Jul 4;8:59. doi: 10.1186/s13550-018-0399-z (PMC6031550; doi:10.1186/s13550-018-0399-z)
Supplement: Supplementary file 1 — Figures S1–S9 and Tables S1–S6. (PDF 14174 kb) [file 13550_2018_399_MOESM1_ESM.pdf]

Discriminating Radiation Necrosis from Recurrent Tumor with $[^{18}F]$PARPi and Amino Acid PET in Mouse Models

Supporting Information

Patrick L. Donabedian[1], Susanne Kossatz[1], John A. Engelbach[2], Stephen A. Jannetti[1,4,5], Brandon Carney[1,4,6], Robert J. Young[1,8], Wolfgang Weber[1,7], Joel R. Garbow[2,3], Thomas Reiner[1,8]

[1] Department of Radiology, Memorial Sloan-Kettering Cancer Center, New York, New York, United States of America

[2] Department of Radiology, Washington University, St. Louis, Missouri, United States of America

[3] Alvin J. Siteman Cancer Center, Washington University, St. Louis, Missouri, United States of America

[4] Department of Chemistry, Hunter College of the City University of New York, New York, New York, United States of America

[5] Ph.D Program in Biochemistry, Graduate Center of the City University of New York, New York, New York, United States of America

[6] Ph.D Program in Chemistry, Graduate Center of the City University of New York, New York, New York, United States of America

[7] Molecular Pharmacology and Chemistry Program, Memorial Sloan-Kettering Cancer Center, New York, New York, United States of America

[8] Department of Radiology, Weill Cornell Medical College, New York, New York, United States of America

***Corresponding author:**
Thomas Reiner, Ph.D.
Department of Radiology, MSK
1275 York Avenue
New York, NY 10065
Phone: 646-888-3461
Fax: 646-422-0408
Email: reinert@mskcc.org

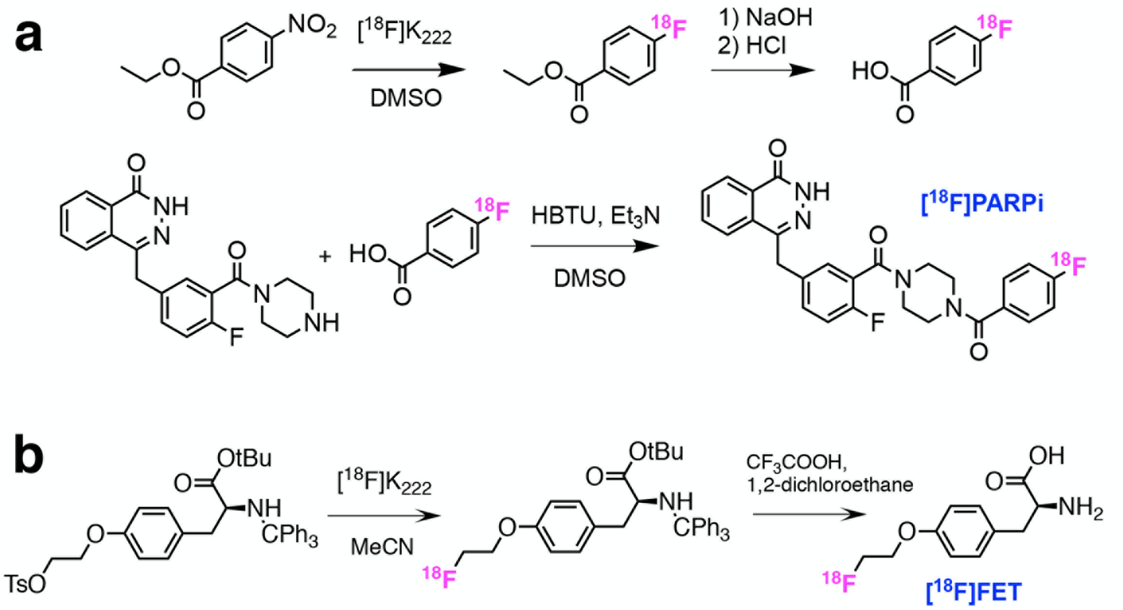

Fig. S1 Schemes for the radiosyntheses of $[^{18}F]$PARPi and $[^{18}F]$FET. (a) Radiosynthesis of $[^{18}F]$PARPi was accomplished in a two-step labeling starting with nucleophilic $[^{18}F]$fluorination of a prosthetic group followed by amide bond coupling to a PARP inhibitor scaffold; (b) radiosynthesis of $[^{18}F]$FET was accomplished by direct fluorination of a tosylated precursor followed by deprotection. Both tracers were purified by HPLC.

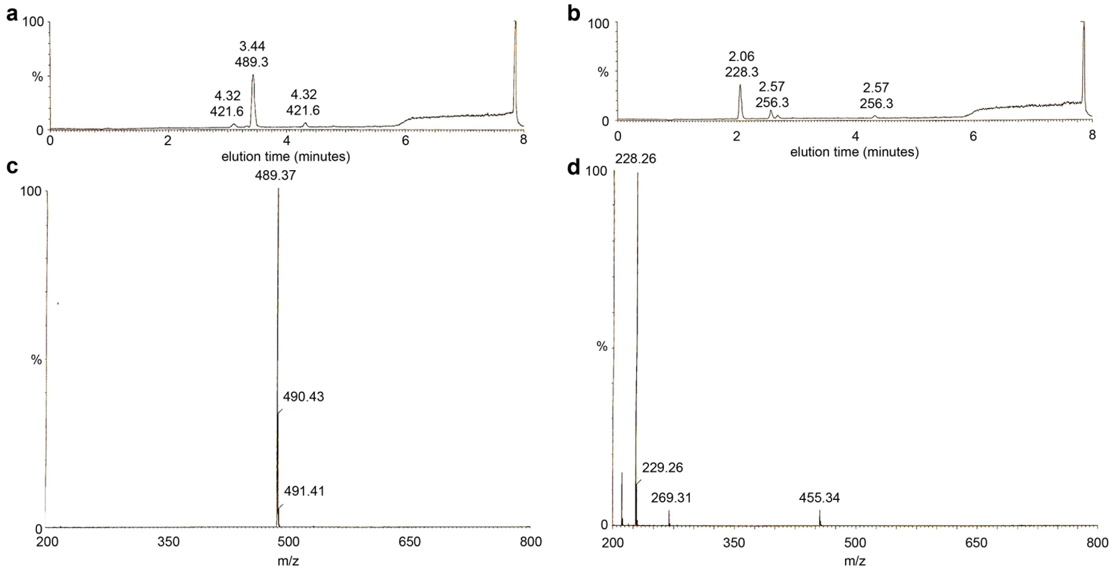

Fig. S2 Verification of cold standard chemical identity: (a) mass chromatogram for $[^{19}F]$PARPi; (b) mass chromatogram for $[^{19}F]$FET; (c) mass spectrum for $[^{19}F]$PARPi; (d) mass spectrum for $[^{19}F]$FET. For $[^{19}F]$PARPi: expected $[M+H]^+$ 489.17, found 489.37. For $[^{19}F]$FET: expected $[M+H]^+$ 228.1, found 228.26.

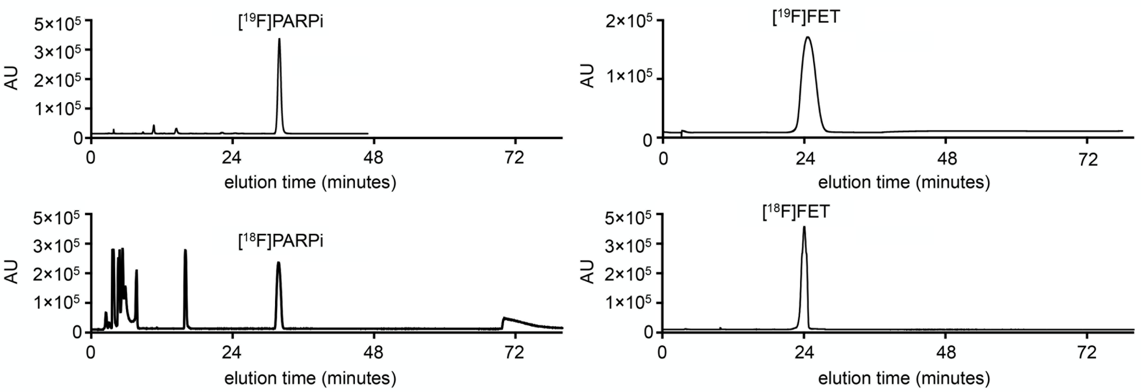

Fig. S3 Verification of cold standard coelution with radiopharmaceutical. Cold standard HPLC chromatogram (UV280, top) and purification HPLC chromatogram (radio-HPLC, bottom) for [^{18}F]PARPi (left) and [^{18}F]FET (right). [24]

Table S1 short tandem repeat fingerprinting results.

% Match	Cell No.	Cell name	Locus names								
			D5S818	D13S317	D7S820	D16S539	VWA	TH01	AM	TPOX	CSF1PO
	U251		11	10,11	10,12	12	16,18	9.3	X	8	12,13
**94%**	IFO5028 5	U-251 MG (KO)	11,11	10,11	10,12	12,12	16,18	9.3,9.3	X,X	8,8	11,12

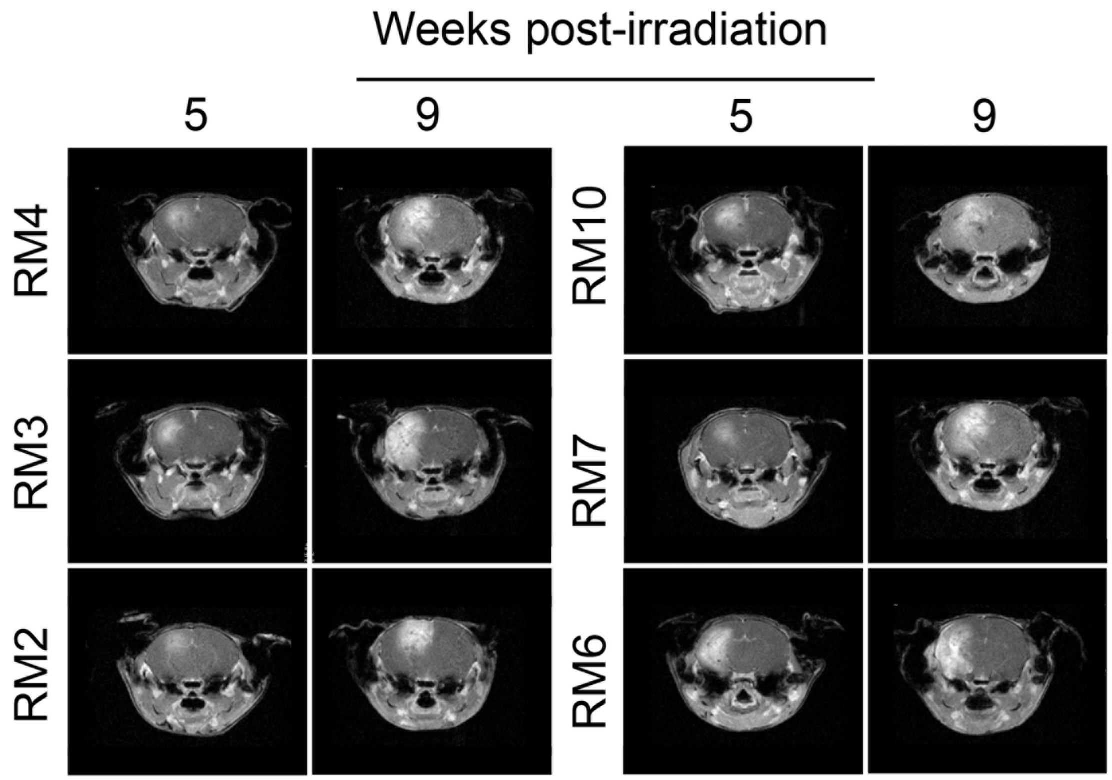

Fig. S4 Course of experimental radiation necrosis lesions by DCE-MR.

Table S2. Complete data on PET VOIs for mice with experimental radiation necrosis injected with [^{18}F]FET.

Subject	Region	%ID/cc$_{mean}$	%ID/cc$_{SD}$	%ID/cc$_{min}$	%ID/cc$_{max}$	max/max	mean/mean	max/mean
RM2	necrosis	9.99	1.03	6.90	13.31	2.19	2.38	3.17
	CL	4.20	0.85	1.93	6.09			
RM3	necrosis	11.23	0.92	8.55	13.09	1.96	2.12	2.47
	CL	5.30	0.45	4.29	6.69			
RM4	necrosis	5.93	0.88	3.55	8.61	2.22	2.89	4.20
	CL	2.05	0.68	0.93	3.88			

Complete data on PET VOIs for mice with experimental radiation necrosis injected with [^{18}F]PARPi.

Subject	Region	%ID/cc$_{mean}$	%ID/cc$_{SD}$	%ID/cc$_{min}$	%ID/cc$_{max}$	max/max	mean/mean	max/mean
RM10	necrosis	0.27	0.18	0.00	1.41	1.07	1.60	8.33
	CL	0.17	0.12	0.00	1.31			
RM3	necrosis	0.22	0.16	0.00	1.32	0.76	1.45	8.56
	CL	0.15	0.13	0.00	1.74			
RM4	necrosis	0.19	0.15	0.00	1.22	1.22	1.38	8.97
	CL	0.14	0.11	0.00	1.00			

CL = contralateral.

Table S3. Complete data on PET VOIs for U251 tumor-bearing mice injected with [^{18}F]FET.

Subject	Region	%ID/cc$_{mean}$	%ID/cc$_{SD}$	%ID/cc$_{min}$	%ID/cc$_{max}$	max/max	mean/mean	max/mean	CEA (mm^3)
TM1*	tumor	2.70	0.48	1.08	5.32	1.10	0.97	1.90	0
	CL	2.79	0.51	1.13	4.82				
TM2	tumor	1.73	0.54	0.27	4.64	1.17	1.08	2.90	7.3
	CL	1.60	0.37	0.47	3.96				
TM3*	tumor	1.76	0.43	0.50	4.56	1.24	0.98	2.54	0
	CL	1.80	0.43	0.75	3.69				
TM4*	tumor	1.81	0.45	0.74	4.82	1.11	0.95	2.52	1.2
	CL	1.91	0.42	0.72	4.34				
TM5	tumor	1.96	0.55	0.69	4.76	1.23	1.07	2.60	6.4
	CL	1.83	0.44	0.67	3.87				
TM6	tumor	2.02	0.46	0.81	3.86	0.94	1.05	2.01	6.6
	CL	1.92	0.42	0.43	4.11				
TM7	tumor	3.85	1.96	1.31	11.24	1.92	1.51	4.42	15.9
	CL	2.55	0.57	0.57	5.87				
TM8	tumor	2.46	0.54	0.76	5.51	1.28	1.10	2.46	8.5
	CL	2.25	0.50	0.55	4.30				
TM9	tumor	4.44	2.53	1.08	15.16	2.00	1.71	5.83	23.8
	CL	2.60	0.64	0.46	7.59				

CL = contralateral; CEA = contrast enhancing area (based on single-frame postcontrast MRI analysis).
*mouse was excluded from statistical analysis of PET data based on CEA < 1.5 mm^3.

Table S4. Complete data on PET VOIs for U251 tumor-bearing mice injected with [^{18}F]PARPi.

Subject	Region	%ID/cc$_{mean}$	%ID/cc$_{SD}$	%ID/cc$_{min}$	%ID/cc$_{max}$	max/max	mean/mean	max/mean	CEA (mm^3)
TM1*	tumor	0.11	0.07	0.00	0.66	0.97	0.68	4.04	0
	CL	0.16	0.09	0.00	0.68				
TM2	tumor	0.14	0.11	0.00	0.84	1.59	1.41	8.24	7.3
	CL	0.10	0.07	0.00	0.53				
TM3*	tumor	0.11	0.10	0.00	0.85	1.17	0.81	6.04	0
	CL	0.14	0.09	0.00	0.72				
TM4*	tumor	0.15	0.11	0.01	0.74	0.99	1.13	5.47	1.2
	CL	0.14	0.09	0.00	0.74				
TM5	tumor	0.18	0.18	0.00	1.36	1.63	1.22	9.05	6.4
	CL	0.15	0.10	0.00	0.83				
TM6	tumor	0.14	0.10	0.00	0.87	1.08	1.08	6.50	6.6
	CL	0.13	0.09	0.00	0.80				
TM7	tumor	0.50	0.56	0.00	2.82	2.66	3.34	18.86	15.9
	CL	0.15	0.13	0.00	1.06				
TM8	tumor	0.12	0.11	0.00	0.85	1.36	1.24	8.78	8.5
	CL	0.10	0.08	0.00	0.63				
TM9	tumor	0.83	0.85	0.00	5.35	3.08	4.41	28.41	23.8
	CL	0.19	0.18	0.00	1.73				
TM10	tumor	0.30	0.37	0.00	2.36	2.19	1.97	15.54	8.10
	CL	0.15	0.12	0.00	1.07				

CL = contralateral; CEA = contrast enhancing area (based on single-frame postcontrast MRI analysis).
*mouse was excluded from statistical analysis of PET data based on CEA < 1.5 mm^3.

Table S5. Complete data on PET VOIs for treatment naïve mice injected with [^{18}F]FET.

Subject	Region	%ID/cc$_{mean}$	%ID/cc$_{SD}$	%ID/cc$_{min}$	%ID/cc$_{max}$	max/max	mean/mean	max/mean
H5	right	0.98	0.58	0.02	6.43	1.22	1.06	6.99
	left	0.92	0.52	0.02	5.28			
H6	right	3.73	1.43	0.40	12.22	0.98	1.02	3.34
	left	3.66	1.36	0.64	12.42			
H7	right	3.11	1.05	0.22	8.76	0.81	0.99	2.80
	left	3.12	1.01	0.31	10.86			
H8	right	2.27	1.09	0.05	9.12	1.15	0.90	3.62
	left	2.52	1.07	0.17	7.93			

Complete data on PET VOIs for treatment naïve mice injected with [^{18}F]PARPi.

Subject	Region	%ID/cc$_{mean}$	%ID/cc$_{SD}$	%ID/cc$_{min}$	%ID/cc$_{max}$	max/max	mean/mean	max/mean
H1	right	0.21	0.13	0.01	1.04	1.02	1.04	5.11
	left	0.20	0.12	0.01	1.01			
H2	right	0.18	0.10	0.01	0.81	1.02	1.12	4.91
	left	0.16	0.09	0.01	0.79			
H3	right	0.17	0.11	0.00	1.10	1.05	1.09	6.85
	left	0.16	0.12	0.00	1.04			
H4	right	0.20	0.11	0.00	0.90	1.02	1.22	5.38
	left	0.17	0.11	0.00	0.88			

CL = contralateral.

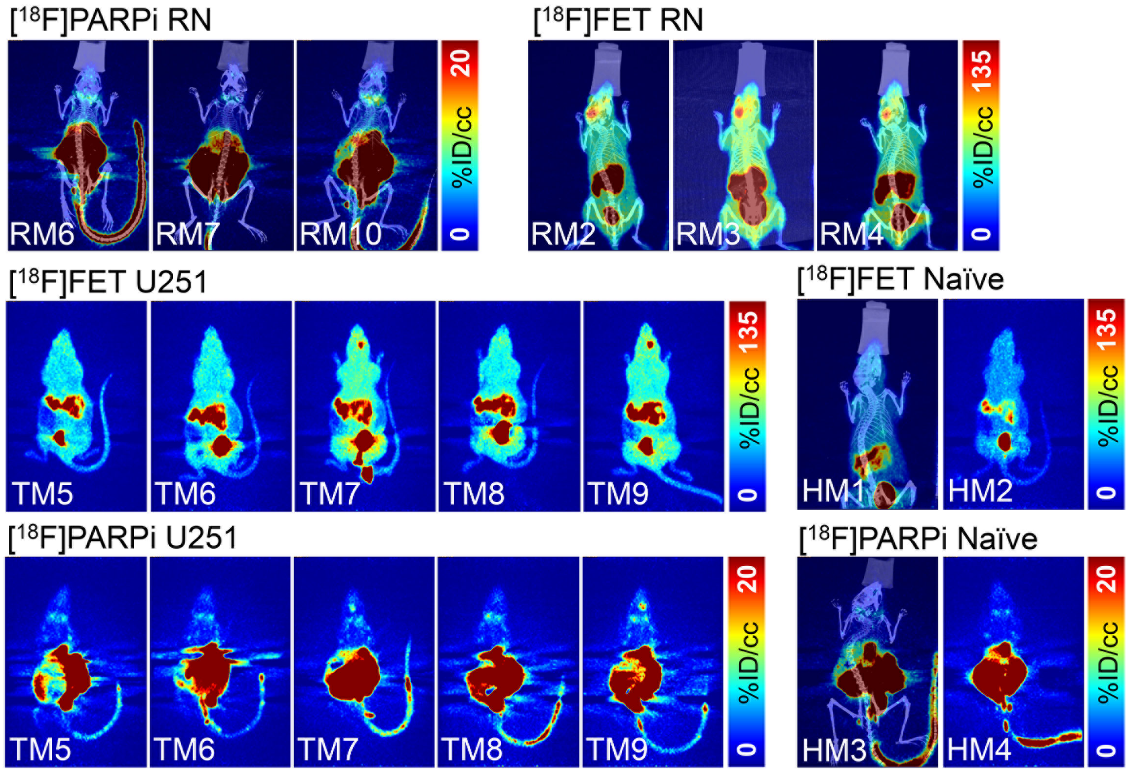

Fig. S5 PET and PET/CT maximum intensity projections.

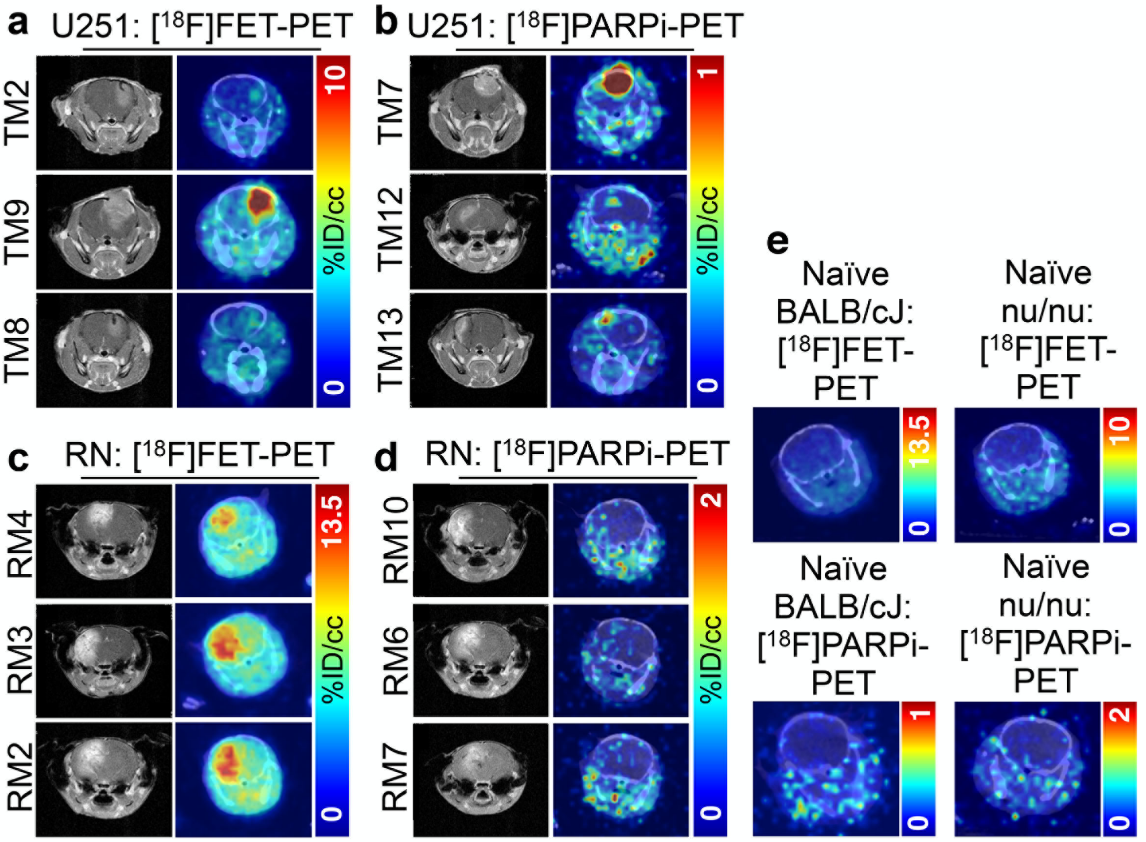

Fig. S6 Additional DCE-MR and PET/CT images. (a) DCE-MR and fused PET/CT transaxial slices for U251 tumor-bearing mice injected with [^{18}F]FET; (b) DCE-MR and fused PET/CT transaxial slices for U251 tumor-bearing mice injected with [^{18}F]PARPi; (c) DCE-MR and fused PET/CT transaxial slices for irradiated mice injected with [^{18}F]FET; (d) DCE-MR and fused PET/CT transaxial slices for irradiated mice injected with [^{18}F]PARPi; (e) fused PET/CT transaxial slices for treatment naïve mice injected with [^{18}F]FET (top row) and [^{18}F]PARPi (bottom row).

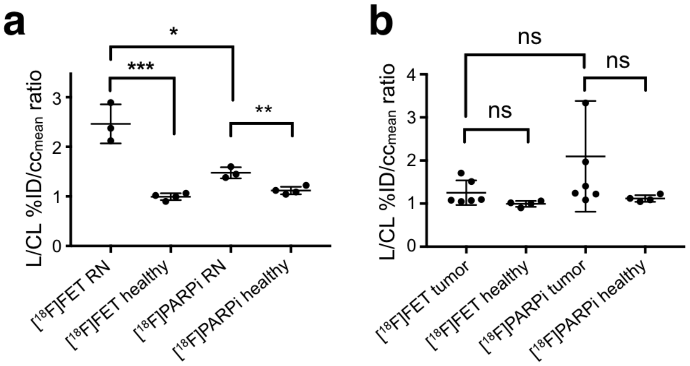

Fig. S7 Quantification of mean uptake ratios for PET VOIs. (a) Lesioned-to-contralateral hemisphere $\%ID/cc_{mean}$ ratios for mice in radiation necrosis and healthy groups. (b) Lesioned-to-contralateral hemisphere $\%ID/cc_{mean}$ ratios for mice in tumor and healthy groups. One data point is outside the axis limits. *significant at $p<0.05$; **significant at $p<0.005$; ***significant at $p<0.001$; all unpaired t-tests except for within tumor group which used a paired t-test.

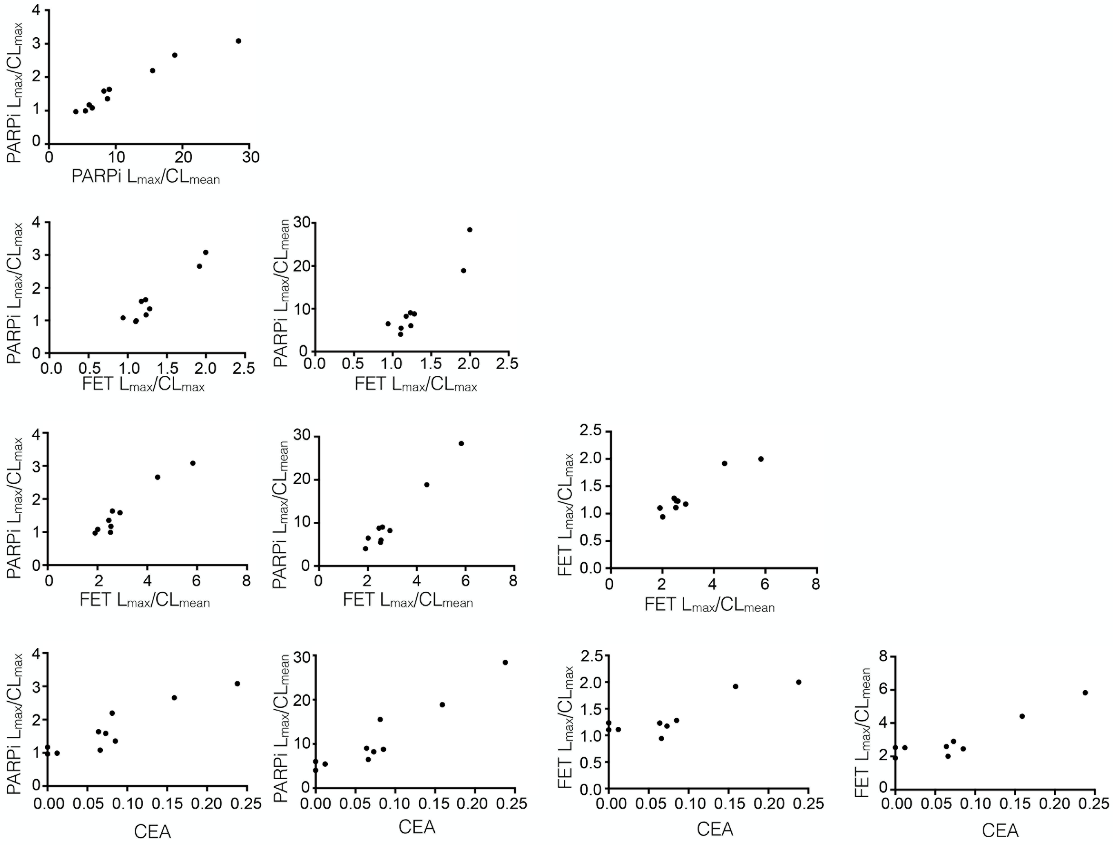

Fig. S8 Correlation of contrast-enhancing area (CEA) on DCE-MR to PARPi-PET and FET-PET L_{max}/CL_{max} and L_{max}/CL_{mean} %ID/cc ratios.

Table S6 Matrix of Pearson's r and p-values for correlation of contrast-enhancing area on DCE-MR to PET L_{max}/CL_{max} and L_{max}/CL_{mean} uptake ratios.

	PARPi L_{max}/CL_{max}	PARPi L_{max}/CL_{mean}	FET L_{max}/CL_{max}	FET L_{max}/CL_{mean}	CEA
PARPi L_{max}/CL_{max}		0.98	0.96	0.97	0.92
PARPi L_{max}/CL_{mean}	0.98		0.94	0.98	0.95
FET L_{max}/CL_{max}	0.96	0.94		0.95	0.87
FET L_{max}/CL_{mean}	0.97	0.98	0.95		0.92
CEA	0.92	0.95	0.87	0.92	
	PARPi L_{max}/CL_{max}	PARPi L_{max}/CL_{mean}	FET L_{max}/CL_{max}	FET L_{max}/CL_{mean}	CEA
PARPi L_{max}/CL_{max}		1.61×10^{-6}	5.02×10^{-5}	2.03×10^{-5}	1.54×10^{-4}
PARPi L_{max}/CL_{mean}	1.61×10^{-6}		1.64×10^{-4}	2.68×10^{-6}	3.29×10^{-5}
FET L_{max}/CL_{max}	5.02×10^{-5}	1.64×10^{-4}		8.80×10^{-5}	2.42×10^{-3}
FET L_{max}/CL_{mean}	2.03×10^{-5}	2.68×10^{-6}	8.80×10^{-5}		5.16×10^{-4}
CEA	1.54×10^{-4}	3.29×10^{-5}	2.42×10^{-3}	5.16×10^{-4}	

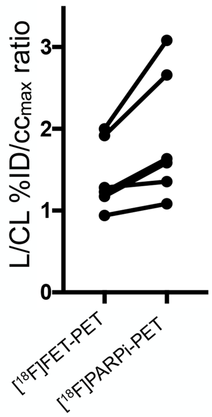

Fig. S9 Tumor/contralateral $\%ID/cc_{max}$ ratios for U251 tumor-bearing mice imaged on successive days with both [^{18}F]PARPi and [^{18}F]FET. Connecting lines are drawn to indicate paired relationships.
